# Early Divergent Cardiac Adaptation After Hematopoietic Stem Cell Transplantation: A Multimodal Echocardiographic and Electrocardiographic Study

**DOI:** 10.3390/diagnostics16101423

**Published:** 2026-05-07

**Authors:** Çetin Alak, Fazil Çağrı Hunutlu, Gokhan Ocakoglu, Nuray Mammadova, Zeynep Kumral, Vildan Ozkocaman, Fahir Ozkalemkas, Dilek Yeşilbursa

**Affiliations:** 1 Department of Cardiology, Faculty of Medicine, Bursa Uludag University, Bursa 16059, Türkiye; nuraymammadova88@gmail.com (N.M.); dilekyb@uludag.edu.tr (D.Y.); 2 Division of Hematology, Department of Internal Medicine, Bursa Sehir Training and Research Hospital, University of Health Sciences, Bursa 16250, Türkiye; fazilhunutlu@gmail.com; 3 Department of Biostatistics, Faculty of Medicine, Bursa Uludag University, Bursa 16059, Türkiye; gocakoglu@gmail.com; 4 Department of Cardiology, Unye State Hospital, Ordu 52300, Türkiye; zeynepkumral@gmail.com; 5 Division of Hematology, Department of Internal Medicine, Faculty of Medicine, Bursa Uludag University, Bursa 16059, Türkiye; vildanoz@uludag.edu.tr (V.O.); fahir@uludag.edu.tr (F.O.)

**Keywords:** hematopoietic stem cell transplantation, left atrial strain, global longitudinal strain, right ventricular function, electrocardiography, cardiac remodeling

## Abstract

**Background/Objectives**: Hematopoietic stem cell transplantation (HSCT) exposes patients to cardiovascular stress through inflammation, metabolic disturbances, and prior cardiotoxic therapies. Although overt dysfunction is uncommon early after transplantation, subclinical cardiac adaptation remains poorly defined. **Methods**: We evaluated early electrical and mechanical cardiac responses after HSCT using integrated electrocardiographic (ECG) and echocardiographic assessment. In this prospective cohort study, patients underwent paired pre-transplant and early post-transplant (3–6 months) ECG and comprehensive echocardiography, including tissue Doppler and speckle-tracking analyses of atrial, ventricular, and right heart function. **Results**: Patients were stratified into multiple myeloma (MM) and non-MM subgroups. ECG voltage increased after HSCT, particularly in MM patients, without changes in left ventricular (LV) mass, geometry, or global systolic indices, suggesting electrical–structural dissociation. Left atrial (LA) reservoir strain decreased despite stable atrial volumes. Diastolic indices showed selective modulation, including a group-time interaction in the lateral e′/a′ ratio without elevated filling pressures. Subgroup analyses suggested divergent myocardial responses, with numerical global longitudinal strain (GLS) improvement in MM patients and reduced LV deformation and right ventricular (RV) fractional area change in non-MM patients. **Conclusions**: Early cardiac responses after HSCT were heterogeneous and compartment-specific, supporting multiparametric imaging for detection of subclinical cardiac adaptation.

## 1. Introduction

HSCT is an established therapy for hematologic malignancies and bone marrow failure syndromes, improving survival in conditions such as multiple myeloma, lymphomas, and leukemias [1]. However, HSCT is associated with a substantial cardiovascular burden related to cumulative cardiotoxic therapies, high-dose conditioning regimens, systemic inflammation, endothelial injury, and marked hemodynamic stress during the peri-transplant period [2,3,4,5]. Current ESC cardio-oncology guidelines therefore identify HSCT recipients as a high-risk population requiring structured cardiovascular surveillance [6].

Early cardiovascular complications, including arrhythmias, heart failure, and subclinical ventricular dysfunction, contribute to post-transplant morbidity, particularly within the first months after HSCT [7,8,9,10,11]. Although long-term cardiotoxicity is well described, early cardiac remodeling during the 3–6-month post-transplant period remains poorly characterized. Previous studies have largely relied on conventional markers such as left ventricular ejection fraction (LVEF) and circulating biomarkers [12,13,14,15,16]. The cardiovascular effects observed during the peri-transplant period are multifactorial and include direct myocardial injury related to conditioning regimens, systemic inflammatory activation, endothelial dysfunction, and significant hemodynamic fluctuations due to volume shifts, anemia, and infection risk. The peri-transplant period generally refers to the interval spanning conditioning therapy through the early post-transplant phase, typically encompassing the first weeks to months after HSCT, when these factors are most pronounced.

Therefore, we performed a prospective multimodal cardiac evaluation using ECG and comprehensive echocardiography before transplantation and at early follow-up (3–6 months) to characterize compartment-specific cardiac remodeling after HSCT and explore disease-specific adaptation patterns.

## 2. Materials and Methods

### 2.1. Study Design and Population

This single-center prospective observational cohort study evaluated early structural and functional cardiac remodeling over 3–6 months in adult patients with hematologic malignancies undergoing HSCT at a tertiary academic center. All participants underwent baseline transthoracic echocardiography (TTE) and 12-lead ECG prior to transplantation, with repetition of the same protocol at 3–6 months post-transplant.

### 2.2. Eligibility Criteria and Study Cohort

Eligible patients were ≥18 years with histologically or cytologically confirmed hematologic malignancy, preserved baseline LVEF (≥50%), and complete baseline and follow-up TTE and ECG data. Patients were excluded if they had inadequate TTE image quality, prior HSCT, or baseline LVEF <50%. No patients with known infiltrative cardiomyopathies, including cardiac amyloidosis, were identified in the study cohort. These conditions were not present among included patients and are recognized as potential confounders of myocardial structural and functional assessments. Patients followed at external centers were not included as complete follow-up echocardiographic data at the predefined time points could not be consistently obtained, which was required for standardized paired analysis.

**Figure 1:**
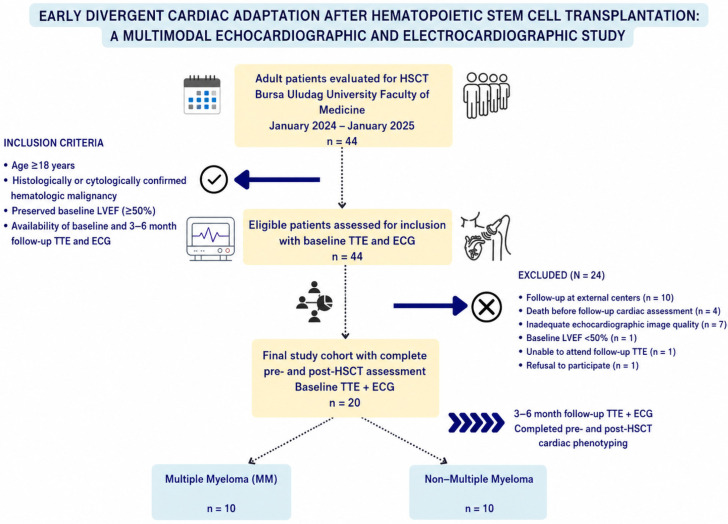
Study flow diagram.

Between January 2024 and January 2025, 44 consecutive adults were screened for HSCT. Twenty-four patients were excluded due to follow-up at external centers (n = 10), death before follow-up (n = 4), baseline LVEF < 50% (n = 1), inability to attend follow-up imaging (n = 1), refusal to participate (n = 1), or inadequate image quality (n = 7). The final cohort comprised 20 patients with complete paired echocardiographic and ECG assessments (Figure 1).

### 2.3. Ethics and Consent

The study protocol was approved by the Bursa Uludag University Faculty of Medicine’s Ethics Committee (Approval No: 2023-27/49) Bursa, Türkiye and conducted in accordance with the Declaration of Helsinki. Written informed consent was obtained from all participants. Analyses were performed on anonymized imaging and ECG data.

### 2.4. Transplant Conditioning and Post-Transplant Management

Conditioning regimens were selected according to hematologic diagnosis and institutional protocols: busulfan–cyclophosphamide for acute leukemia undergoing allogeneic HSCT, BEAM for lymphoma, and high-dose melphalan (200 mg/m^2^) for MM undergoing autologous HSCT.

Post-transplant granulocyte-colony stimulating factor was routinely initiated on day +5 in autologous HSCT recipients. In allogeneic HSCT, graft-versus-host disease prophylaxis consisted of cyclosporine-A and methotrexate, according to standard practice.

### 2.5. Transthoracic Echocardiographic Assessment

TTE examinations were performed using a GE Vivid E95 system (GE Healthcare, Horten, Norway) with a phased-array transducer under standardized resting conditions. Baseline imaging was obtained immediately before HSCT and repeated at 3–6 months, using an identical protocol in accordance with ASE and EACVI recommendations [17].

Standard two-dimensional, M-mode, Doppler, and speckle-tracking datasets were acquired in the left lateral decubitus position and stored in DICOM format. Speckle-tracking analyses were performed at frame rates of 60–90 frames/s using vendor-independent software (TOMTEC Imaging Systems, Munich, Germany).

LVEF was calculated using the biplane Simpson method. LV dimensions, wall thickness, relative wall thickness, and geometry indices were derived from parasternal long-axis views. LV mass was calculated using ASE-corrected and Penn convention formulas with indexed values. GLS was assessed from apical two-, three-, and four-chamber views, including myocardial-layer strain when tracking quality permitted [18].

All echocardiographic analyses were performed by experienced cardiologists using a standardized acquisition and post-processing protocol, with offline analysis and careful verification of tracking quality. While these measures reduce variability, intra- and inter-observer variability cannot be entirely excluded, particularly for deformation imaging techniques.

### 2.6. Left Atrial Strain Analysis

LA mechanics were assessed using speckle-tracking echocardiography according to EACVI/ASE recommendations. LA endocardial borders were traced in apical four- and two-chamber views, with automated tracking visually inspected and manually adjusted when necessary. Strain curves were R-wave-gated with zero reference at LV end-diastole, and reservoir, conduit, and contraction strain parameters were derived using standardized methodologies [19].

### 2.7. Right Heart and Additional Functional Assessment

RV function was evaluated using fractional area change, RV free-wall longitudinal strain when feasible, and lateral tricuspid annular tissue Doppler velocities (S′, e′, a′), according to guideline recommendations [18,19]. Right atrial dimensions were measured using standard views. All analyses were performed by two experienced cardiologists blinded to clinical diagnosis and study time-point.

### 2.8. Echocardiographic Post-Processing

Strain and volumetric measurements were generated using automated contour detection with manual adjustments to ensure accurate endocardial delineation. Only segments with adequate tracking were included in the analysis.

### 2.9. Electrocardiographic Assessment

Standard 12-lead ECGs were recorded at 25 mm/s and 10 mm/mV in the supine resting position before HSCT and at 3–6-month follow-up. Heart rate, P-wave duration, PR interval, QRS duration, QT/QTc, RV5 and SV1 amplitudes, and Sokolow–Lyon index were obtained from automated measurements and independently verified by two blinded cardiologists (Figure 1).

### 2.10. Statistical Analysis

Continuous variables were tested for normality using the Shapiro–Wilk test. As variables were non-normally distributed, descriptive statistics are presented as median (minimum–maximum). Baseline comparisons between the MM (n = 10) and non-MM (n = 10) groups were performed using the Mann–Whitney U test, with effect sizes reported as rank-biserial correlation coefficients (r) and interpreted as small (≥0.10), medium (≥0.30), or large (≥0.50).

Within-group longitudinal changes between pre-transplant (Pre) and 3–6-month post-transplant (Post) evaluations were assessed using the Wilcoxon signed-rank test. To evaluate the main effects of Time (Pre vs. Post), Group (MM vs. non-MM), and Time × Group interaction effects on echocardiographic and ECG parameters, a non-parametric factorial analysis was performed using the aligned rank transform (ART) procedure implemented in the ARTool package in R (version 4.3.2). Effect sizes were reported as partial eta squared (ηp^2^) and interpreted as small (0.01), medium (0.06), or large (0.14).

To control for cumulative Type I error across multidimensional imaging variables, parameters were grouped into eight predefined physiological domains, and a Benjamini–Hochberg false discovery rate (FDR) correction was applied within each domain. Associations between structural and electrical remodeling were assessed using delta change values (Δ = Post − Pre) and Spearman’s rank correlation.

Data management and descriptive analyses were performed in IBM SPSS Statistics version 26.0, while domain-corrected longitudinal and interaction analyses were conducted in R (version 4.3.2). Two-sided *p* < 0.05 (or FDR-adjusted q < 0.05) was considered statistically significant.

## 3. Results

### 3.1. Study Population and Statistical Approach

Baseline demographic, clinical, and transplant-related characteristics of the cohort are summarized in Table 1. Clinical, ECG, and TTE data obtained at baseline (pre-transplant) and early follow-up (3–6 months) from MM and non-MM participants (n = 20) were analyzed. As variables were non-normally distributed, data are presented as median (minimum–maximum). To control for multiple comparisons, a domain-based Benjamini–Hochberg false discovery rate (FDR) correction was applied for longitudinal analyses (Table 1, Table 2, Table 3 and Table 4).

### 3.2. Baseline Characteristics

At baseline, the MM group showed longer ECG P-wave duration (*p* = 0.041; r = 0.46), lower RV FAC (*p* = 0.041; r = 0.46), and lower stroke index (*p* = 0.023; r = 0.51) compared with the non-MM group. Diastolic indices also differed, with higher septal and lateral a′ velocities but a lower lateral e′/a′ ratio in the MM group (all *p* < 0.05), suggesting distinct pre-transplant atrial–ventricular coupling.

### 3.3. Within-Group Changes

#### 3.3.1. Multiple Myeloma

In the MM group, BMI decreased (*p* = 0.024) and ECG RV5–SV1 voltage increased during follow-up (*p* = 0.047). Myocardial GLS in the apical two-chamber view changed significantly (*p* = 0.013), whereas conventional systolic indices showed modest deterioration with reduced EF (A4C) and increased automated end-systolic volume index (both *p* = 0.047).

LA mechanics showed the most prominent changes, with reductions in LA reservoir and contractile strain (both *p* = 0.013). Decreases were also observed in septal a′ velocity, lateral S′ velocity, and RV lateral a′ velocity (all *p* < 0.05).

#### 3.3.2. Non–Multiple Myeloma

In the non-MM group, significant reductions in LVIDd and LVIDs suggested geometric remodeling without global systolic deterioration. Regional deformation analysis showed significant changes in myocardial GLS in the apical four- and three-chamber views (both *p* < 0.05). Tissue Doppler analysis revealed reductions in lateral e′ velocity (*p* = 0.009) and the lateral e′/a′ ratio (*p* = 0.047), indicating early impairment of diastolic relaxation.

### 3.4. Longitudinal Analysis

#### 3.4.1. Time × Group Interaction

After FDR correction, a significant interaction persisted only for the lateral e′/a′ ratio (*p* = 0.003; q = 0.031; ηp^2^ = 0.40), indicating a differential diastolic response between MM and non-MM groups.

#### 3.4.2. Main Effects

Significant temporal changes included decreases in BMI (*p* = 0.008; q = 0.025) and changes in myocardial deformation parameters, particularly AutoGLS in A2C and A4C views (both q < 0.05). LA reservoir and contractile strain rates also showed significant temporal changes (q = 0.008).

For the main effect of group, persistent differences were observed in ECG P-wave duration (q = 0.043) and RV FAC (q = 0.049).

### 3.5. Correlation Analysis

Spearman’s correlation analysis showed no significant associations between ΔRV5+SV1 voltage and changes in LV mass across multiple calculation methods (all *p* > 0.70). Further results are provided in Supplementary Tables S1 and S2.

## 4. Discussion

### 4.1. Electrical–Structural Dissociation: ECG Voltage and Left Ventricular Mass

Following HSCT, within-group analyses demonstrated a modest but significant increase in surface ECG voltage, most prominently reflected by higher RV5+SV1 amplitude in the MM subgroup. This raised the question of whether post-transplant voltage augmentation reflects structural myocardial regression, particularly reduced LV mass, or an electrical phenomenon independent of macroscopic remodeling [7,20].

To investigate this, we examined the relationship between ECG voltage changes and multiple echocardiographic LV mass formulations and indexing strategies. Despite the consistent numerical increase in ECG voltage, no correlation was observed between ΔRV5+SV1 voltage and cube-derived, body surface area-indexed, height-indexed, or Penn-derived LV mass across the cohort, including both MM and non-MM subgroups [7].

Although a modest reduction in indexed LV mass was observed at the group level, this decrease occurred alongside a significant decline in BMI. This parallel change suggests that post-transplant catabolic and anthropometric alterations may affect indexed geometric measurements rather than represent true regression of myocardial mass.

Changes in ECG voltage showed no meaningful association with LV dimensions, wall thickness, global systolic performance, or myocardial deformation parameters.

Taken together, these observations indicate a dissociation between electrical findings and structural myocardial characteristics following HSCT. In other words, the early increase in ECG voltage does not appear to reflect changes in macroscopic ventricular geometry.

Previous investigations have also reported a weak relationship between surface ECG voltage and true LV mass, emphasizing that voltage changes should be interpreted cautiously, and not assumed to represent structural myocardial recovery [7,21].

Collectively, these observations suggest that early post-transplant ECG voltage augmentation likely reflects transient functional adaptation related to loading conditions, systemic inflammation, and autonomic changes rather than structural myocardial remodeling [4,5,21,22,23].

### 4.2. Left Atrial Mechanics After HSCT

Initial within-group comparisons revealed modest pre–post differences in several LA deformation parameters, predominantly in the MM subgroup. These included reductions in LASr ED, LAScd ED, and LASr AC, suggesting a potential early modulation of atrial functional phases after HSCT.

When longitudinal modeling was performed—including the effects of Group, Time, and Group × Time interaction with FDR correction—only LASr ED maintained a significant main effect of time, while the remaining atrial deformation indices no longer reached statistical significance. This suggests that the paired changes observed in the preliminary analyses were not consistently maintained in the multivariable longitudinal framework.

LA volumes and indexed LA volumes remained largely unchanged throughout follow-up, and no significant interaction between group and time was identified for either structural or deformation-related parameters. Overall, this pattern points to a selective atrial response in which attenuation of end-diastole-referenced LA reservoir strain appears to be the most consistent signal, rather than evidence of global atrial dysfunction or structural remodeling.

Previous cardio-oncology studies have described LA reservoir strain as a sensitive indicator of early cardiovascular stress, frequently preceding detectable atrial enlargement or overt diastolic dysfunction. In patients undergoing HSCT, alterations in atrial strain have been attributed mainly to transient changes in loading conditions, myocardial–atrial coupling, and systemic inflammatory responses, rather than to permanent atrial structural remodeling [5,10,13,17,24].

### 4.3. Diastolic Parameters: Selective and Heterogeneous Changes

Initial within-group comparisons identified pre–post differences in several tissue Doppler–derived parameters following HSCT—most notably, reductions in septal a′ and septal S′ velocities in the MM subgroup. Considering the contribution of these velocities to late diastolic filling, these observations warranted further evaluation using longitudinal modeling.

When the analysis incorporated the effects of Group, Time, and Group × Time interaction with FDR correction, neither septal a′ nor septal S′ maintained a significant main effect of time, and no consistent interaction pattern emerged. Among the conventional diastolic indices, only the lateral e′/a′ ratio showed a significant Group × Time interaction.

Other established indicators of elevated LV filling pressure—including the E/e′ ratio, transmitral inflow velocities, deceleration time, and LA volume indices—did not show meaningful changes during follow-up.

The observed alterations in a′ velocity and e′/a′ ratio occurred without changes in LA size, transmitral flow patterns, or filling pressure surrogates, arguing against overt diastolic dysfunction in the early post-transplant period. Instead, these findings suggest selective modulation of late diastolic mechanics, likely reflecting a transient shift in the contribution of atrial contraction to ventricular filling.

Within current guideline frameworks, isolated tissue Doppler changes without structural or hemodynamic abnormalities should be interpreted cautiously. Accordingly, the FDR-sensitive alterations observed in our cohort most likely represent early functional adaptations of diastolic filling dynamics rather than clinically meaningful diastolic dysfunctions after HSCT [4,5,6,11,15,25].

### 4.4. Left Ventricular Systolic and Myocardial Deformation: Divergent Patterns by Disease Subtype

The comparison between MM and non-MM groups was chosen to explore potential differences in early post-HSCT cardiac adaptation across clinically distinct transplant populations with different disease characteristics and treatment exposures. Analyses of LV systolic performance and myocardial deformation revealed divergent response patterns between patients with multiple myeloma (MM) and those with non-MM diagnoses, suggesting disease- and exposure-related myocardial adaptation in the early post-HSCT period.

In within-group analyses, MM patients showed modest reductions in visually derived EF (A4C) and an increase in end-systolic volume index; however, these changes did not persist after longitudinal modeling with multiple testing correction. No significant Time or Group × Time effects were observed for biplane EF, stroke volume, or end-diastolic volume indices. In contrast, myocardial deformation analysis demonstrated numerical improvement in myocardial-layer GLS in the apical two-chamber view, indicating subtle modulation of myocardial contractile mechanics rather than progressive systolic dysfunction or structural remodeling [16,20,21,22].

The non-MM subgroup showed a different response pattern characterized by reductions in LV internal diameters together with worsening myocardial-layer GLS in the apical four- and three-chamber views. These deformation changes were accompanied by reductions in lateral e′ velocity and the lateral e′/a′ ratio, while EF, LV volumes, and filling pressure indices remained stable, supporting the presence of subclinical myocardial vulnerability rather than overt systolic or diastolic dysfunction. The non-MM group was heterogeneous and included patients with different hematologic malignancies and conditioning regimens, which may have influenced the observed subgroup patterns. Therefore, these findings should be interpreted cautiously, particularly given the limited sample size and potential differences in treatment-related cardiotoxic exposure. Given the heterogeneous cardiotoxic exposure in the non-MM subgroup, including prior chemotherapy, this deformation-predominant pattern likely reflects early treatment-related myocardial susceptibility. Similar findings have been reported in lymphoma and other non-MM populations, where myocardial deformation abnormalities occur despite preserved EF [5,6,14,26].

Overall, these findings demonstrate a dissociation between conventional systolic indices and myocardial deformation after HSCT, highlighting the incremental value of strain imaging for detecting subtle myocardial responses not captured by LVEF alone [20,21,22].

### 4.5. Right Ventricular Structure and Function After HSCT

Evaluation of RV structure and function demonstrated subtle and heterogeneous changes after HSCT, predominantly affecting tissue Doppler and deformation-derived indices rather than conventional global RV parameters. Initial within-group analyses showed mild reductions in lateral tricuspid annular a′ and S′ velocities and RV FAC, particularly in the non-MM subgroup, while RV longitudinal strain parameters showed only modest attenuation.

When longitudinal modeling accounted for the effects of Group, Time, and their interaction with FDR correction, most RV-related parameters—including TAPSE, RV free-wall strain, and RV four-chamber longitudinal strain—did not show a significant temporal effect. RV FAC appeared to reflect a baseline subgroup difference rather than a longitudinal decline, with lower values observed in the non-MM subgroup irrespective of HSCT-related temporal changes. No meaningful Group × Time interaction was identified for RV indices.

Measures of right heart structure, including RV chamber dimensions and right atrial size, remained largely unchanged during follow-up. This stability argues against the presence of overt RV remodeling or progressive right-sided dysfunction in the early post-transplant phase. Overall, the observed pattern points to relatively limited and predominantly subclinical RV involvement following HSCT.

The lower RV functional indices seen in the non-MM subgroup may therefore be related to underlying myocardial susceptibility associated with prior cardiotoxic treatments rather than representing a direct effect of transplantation itself, a concept that aligns with observations reported in recent cardio-oncology literature [5,9,14,20,23].

From a cardio-oncology perspective, echocardiography represents a first-line imaging modality not only for detecting early functional abnormalities but also as a part of a structured diagnostic pathway. In cases with abnormal or inconclusive findings, further multimodality imaging may be required to characterize structural abnormalities, including masses or other pathologies.

## 5. Study Limitations

This study has several limitations. The small sample size (n = 20), derived from a single center, limits statistical power and generalizability, particularly for subgroup analyses. The findings should be interpreted with caution, considered exploratory and hypothesis-generating, and require confirmation in larger multicenter studies with longer follow-up. Second, cardiac evaluation was performed at only two predefined time points, preventing assessment of longer-term trajectories and reversibility of observed changes.

Third, invasive hemodynamic data, cardiopulmonary exercise testing, and systematic biomarker profiling were unavailable, limiting physiological correlation with imaging findings. Fourth, heterogeneity in prior cardiotoxic treatment exposure represents a potential confounder. Finally, deformation and tissue Doppler indices are load-dependent, and variability related to peri-transplant volume status and systemic conditions cannot be excluded. These findings should therefore be interpreted as evidence of functional adaptation rather than definitive structural injury.

## 6. Conclusions

In this prospective multimodal evaluation, early cardiac remodeling after HSCT was characterized by heterogeneous, compartment-selective, and predominantly functional adaptations rather than uniform structural deterioration. Electrical alterations, including increased ECG voltage, were uncoupled from LV mass, geometry, and global systolic performance, indicating early electrical–structural dissociation. These findings suggest early subclinical cardiac changes after HSCT, characterized by heterogeneous and compartment-specific patterns. However, given the small sample size and short follow-up, the results should be considered exploratory and require validation in larger studies.

The most consistent imaging signal was attenuation of LA reservoir strain in the absence of atrial enlargement or elevated filling pressures, suggesting early modulation of atrial–ventricular coupling rather than overt diastolic dysfunction.

Myocardial responses differed by disease subtype: MM patients showed relative stabilization or numerical improvement in deformation indices, whereas non-MM patients exhibited subclinical reductions in LV deformation and selected RV functional parameters, consistent with increased myocardial vulnerability related to prior cardiotoxic exposure.

These findings highlight the limitations of EF-based surveillance and support the incremental value of integrated multiparametric imaging, particularly strain analysis, for detecting early subclinical cardiac adaptation after HSCT.

Future longitudinal multicenter studies are needed to clarify the prognostic significance and long-term implications of these early compartment-specific cardiac changes.

## Figures and Tables

**Table 1:** Baseline demographic, clinical, and transplant-related characteristics of the study cohort (n = 20).

Characteristic	Value
Demographics	
Age, years, median (IQR)	53.5 (49.5–58.5)
Female sex, n (%)	8 (40%)
Height, cm, mean ± SD	168.6 ± 8.5
Weight, kg, mean ± SD	72.5 ± 10.6
BMI, kg/m^2^, mean ± SD	25.6 ± 3.7
Hemodynamic and laboratory profile (pre-HSCT)	
Heart rate, beats/min, mean ± SD	76.7 ± 12.1
Systolic BP, mmHg, mean ± SD	122.7 ± 14.2
Diastolic BP, mmHg, mean ± SD	77.5 ± 10.0
Hemoglobin, g/dL, mean ± SD	11.1 ± 1.9
Creatinine, mg/dL, median (IQR)	0.80 (0.64–1.18)
GFR, mL/min/1.73 m^2^, mean ± SD	87.2 ± 31.1
CRP, mg/L, median (IQR)	3.3 (1.0–9.9)
BNP, pg/mL, median (IQR), n = 17	27.4 (10.6–66.6)
Troponin, ng/L, median (IQR), n = 17	3.0 (0–6.2)
Underlying hematologic disease, n (%)	
Multiple myeloma, n (%)	10 (50%)
Leukemia, n (%)	6 (30%)
Lymphoma, n (%)	3 (15%)
Myelodysplastic syndrome/other, n (%)	1 (5%)
Transplant characteristics, n (%)	
HSCT type	
Autologous, n (%)	13 (65%)
Allogeneic, n (%)	7 (35%)
Allogeneic donor type	
Matched related donor, n (%)	6 (85.7%)
Matched unrelated donor, n (%)	1 (14.3%)
Stem cell source in allogeneic HSCT	
Peripheral	6 (85.7%)
Bone marrow	1 (14.3%)
Anthracycline exposure, n (%)	8 (40%)
Anthracycline cumulative dose, mg/m^2^, median (IQR)	36 (16–105), range 36–400
Total body irradiation exposure, n (%)	1 (5%)
Comorbidities, n (%)	
Hypertension	2 (10%)
Diabetes mellitus	2 (10%)
Coronary artery disease	0 (0%)
Smoking history	12 (60%)
HCT-CI score, median (IQR)	1 (0–2), range 0–6

Abbreviations: BMI, body mass index; BP, blood pressure; HSCT, hematopoietic stem cell transplantation; GFR, glomerular filtration rate; CRP, C-reactive protein; BNP, B-type natriuretic peptide; HCT-CI, Hematopoietic Cell Transplantation–Comorbidity Index; IQR, interquartile range; SD, standard deviation.

**Table 2:** Significant within-group changes observed during the treatment process in multiple myeloma and non-MM groups.

	Pre-HSCT	Post-HSCT	*p*	ES (r)
MM (n = 10)				
BMI, kg/m^2^	26.75 (17.60: 29.40)	25.30 (16.20: 28.10)	0.024	0.50
ECG RV_5_–SV_1_, voltage	2.14 (0.26: 2.76)	2.43 (1.06: 3.04)	0.047	0.44
EF (A4C), %	62.55 (54.90: 65.80)	58.90 (51.30: 67.40)	0.047	0.44
AutoGLS myo (A2C), %	−14.70 (−19.10: −12)	−17.70 (−19.10: −14.20)	0.013	0.56
Auto ESV index (BP), mL/m^2^	21.80 (19.19: 28.63)	27.04 (16.61: 32.04)	0.047	0.44
Mitral R–R interval, ms	714 (579: 943)	852.50 (647: 1158)	0.047	0.44
Mitral CO duration, ms	365 (315: 415)	404 (348: 447)	0.037	0.47
LA sr (ED), 1/s	55.75 (32.10: 66.70)	40 (31.40: 73.10)	0.013	0.56
LA Scd (ED), 1/s	−25.65 (−313: −19.60)	−20.80 (−41: −10.70)	0.021	0.52
LA sr (AC), 1/s	42.60 (28.50: 53)	33.65 (27.40: 54.80)	0.013	0.56
a′ (MV anulus septal), cm/s	12.64 (9.50: 16.33)	11.67 (8.17: 16.03)	0.013	0.56
S′ septal, cm/s	11.05 (7.61: 12.80)	9.09 (7.65: 11.43)	0.021	0.52
RV a′ lateral, cm/s	17.98 (13.79: 23.38)	15.04 (10.02: 21.83)	0.037	0.47
Non-MM (n = 10)				
LVIDd, mm	47 (40: 54)	45.50 (34: 53)	0.015	0.55
LVIDs, mm	32 (26: 41)	29 (23: 41)	0.035	0.47
AutoGLS myo (A4C), %	−19.40 (−21.80: −12.70)	−16.70 (−20.70: −11.90)	0.038	0.49
AutoGLS myo (A3C), %	−18.60 (−21.40: −12.90)	−16.10 (−19.10: −11.90)	0.036	0.53
e′ lateral (MV anulus), cm/s	13.69 (8.54: 22.55)	10.94 (7.68: 22.81)	0.009	0.58
e′/a′ lateral (MV anulus)	1.48 (0.54: 2.66)	0.81 (0.48: 2.39)	0.047	0.44

Abbreviations: BMI, body mass index; ECG, electrocardiography; RV_5_–SV_1_, Sokolow–Lyon voltage (R wave in V_5_ plus S wave in V_1_); EF, ejection fraction; A4C/A2C/A3C, apical four-/two-/three-chamber view; GLS, global longitudinal strain; myo, myocardial layer; ESV, end-systolic volume; BP, biplane method; CO, closure; LA, left atrium; sr, strain rate; ED, end-diastole; AC, atrial contraction; MV, mitral valve; LVIDd, left ventricular internal diameter in diastole; LVIDs, left ventricular internal diameter in systole; RV, right ventricle; S′, systolic tissue Doppler velocity; e′, early diastolic tissue Doppler velocity; a′, late diastolic tissue Doppler velocity; ES, effect size (rank-biserial correlation coefficient).

**Table 3:** Between-group comparison of selected echocardiographic and ECG parameters in MM and non-MM Patients.

			Non-MM			
	Pre-HSCT	Post-HSCT	Pre-HSCT	Post-HSCT	*p*-Value	ES (r)
ECG-P wave duration, ms	115 (99: 120)	118 (79: 157)	108 (100: 114)	110 (93: 118)	0.041	0.46
AutoGLS myo (A4C), %	−18.20 (−20.50: −14.10)	−17.20 (−18.90: −12.50)	−19.40 (−21.80: −12.70)	−16.70 (20.70: −11.90)	0.438	0.18
AutoGLS myo (A2C), %	−14.70 (−19.10: −12)	−17.70 (−19.10: −14.20)	−16.70 (−19.10: −11.60)	−18.70 (−20.50: −13)	0.326	0.23
RV FAC, %	42.60 (30: 60)	42.40 (29.70: 50.90)	52.70 (44.40: 57.30)	48 (35.60: 57.10)	0.041	0.46
LA sr (ED), 1/s	55.75 (32.10: 66.70)	40 (31.40: 73.10)	46.10 (26.30: 62)	42.90 (12.30: 64.20)	0.075	0.40
LA sr (AC), 1/s	42.60 (28.50: 53)	33.65 (27.40: 54.80)	37.05 (24.60: 49.90)	35.20 (25.70: 47.40)	0.063	0.42
a′ lateral (MV anulus), cm/s	13.88 (8.05: 19.26)	10.88 (9.37: 16.75)	9.84 (7.94: 18.90)	12.02 (6.28: 21.09)	0.038	0.47
e′/a′ lateral (MV anulus)	0.78 (0.61: 1.70)	1.06 (0.61: 1.41)	1.48 (0.54: 2.66)	0.81 (0.48: 2.39)	0.045	0.45

ES: effect size. Descriptive summaries are reported as median (minimum: maximum). Baseline comparisons between groups (pre-HSCT) were performed using the Mann–Whitney U test. Effect sizes were computed as $r=|Z|/\sqrt{n}$ (where Z is the standardized test statistic and n is the total sample size; r is reported as absolute magnitude). Interpretation thresholds (Cohen’s guidelines): small 0.10, medium 0.30, large 0.50+. Abbreviations: A2C/A4C, apical two-/four-chamber; AC, atrial contraction; ED, end-diastole; FAC, fractional area change; GLS, global longitudinal strain; LA, left atrium; MV, mitral valve; RV, right ventricle.

**Table 4:** Longitudinal comparison of clinical and echocardiographic changes between Multiple Myeloma and non–Multiple Myeloma groups: Time, Group, and Time × Group interaction analysis.

	**Group**			Time			Group × Time		
	*p*-Value	q-Value	$\mathrm{ES}\;(\eta_{p}^{2}$)	*p*-Value	q-Value	$\mathrm{ES}\;(\eta_{p}^{2}$)	*p*-Value	q-Value	$\mathrm{ES}\;(\eta_{p}^{2}$)
ECG P duration, ms	0.006	0.043	0.35	0.245	0.344	0.07	0.441	0.631	0.03
AutoGLS myo (A4C), %	0.725	0.869	0.01	0.008	0.045	0.35	0.598	0.790	0.02
AutoGLS myo (A2C), %	0.279	0.869	0.07	0.001	0.013	0.47	0.772	0.790	0.01
RV FAC, %	0.006	0.049	0.35	0.111	0.222	0.14	0.641	0.855	0.01
LA sr (ED), 1/s	0.359	0.923	0.05	0.002	0.018	0.42	0.042	0.356	0.21
LA sr (AC), 1/s	0.521	0.923	0.02	0.002	0.018	0.43	0.007	0.115	0.34
e′/a′ lateral (MV anulus)	0.299	0.547	0.06	0.026	0.237	0.25	0.003	0.031	0.40

ES: effect size. Main effects of Group, Time, and their interaction were analyzed using the non-parametric Aligned Rank Transform (ART) test. To control for multiple comparisons, *p*-values for main analyses were adjusted using the Benjamini–Hochberg (FDR) method; *p*-value represents the nominal (raw) *p*-value, and q-value represents the adjusted *p*-value. Effect sizes were computed as partial eta squared ($\eta_{p}^{2}$). Interpretation thresholds (Cohen’s guidelines): small 0.01, medium 0.06, large ≥0.14. Bold values indicate statistical significance (*p* < 0.05). Abbreviations: AC, atrial contraction; A2C/A4C, apical two-/four-chamber; ED, end-diastole; FAC, fractional area change; FDR, false discovery rate; GLS, global longitudinal strain; LA, left atrium; MV, mitral valve; RV, right ventricle.

## Data Availability

The datasets generated and analyzed during the current study are available from the corresponding author on reasonable request, in accordance with participant confidentiality and institutional regulations.

## Supplementary Materials

The following supporting information can be downloaded at: https://www.mdpi.com/article/10.3390/diagnostics16101423/s1, Supplementary Table S1: Full Between-Group Comparison of Pre-HSCT Clinical, Electrocardiographic, and Echocardiographic Parameters in Multiple Myeloma andNon–Multiple Myeloma Patients, Supplementary Table S2: Full Longitudinal Analysis of Clinical and Echocardiographic Parameters in Multiple Myeloma and Non–Multiple Myeloma Patients: Main Effects of Time, Group, and Time × Group Interaction (ART Model).

## Abbreviations

ECG	Electrocardiography
HSCT	Hematopoietic stem cell transplantation
TTE	Transthoracic echocardiography
LV	Left ventricle/left ventricular
RV	Right ventricle/right ventricular
LA	Left atrium/left atrial
GLS	Global longitudinal strain
FAC	Fractional area change
LVEF	Left ventricular ejection fraction
