# Supplementary material for: Early Divergent Cardiac Adaptation After Hematopoietic Stem Cell Transplantation: A Multimodal Echocardiographic and Electrocardiographic Study
Source: Diagnostics (Basel). 2026 May 7;16(10):1423. doi: 10.3390/diagnostics16101423 (PMC13205220; doi:10.3390/diagnostics16101423)
Supplement: Supplementary file 1 [file diagnostics-16-01423-s001.zip › diagnostics-4280333-supplementary.pdf]

Supplementary Table S1. Full Between-Group Comparison of Pre-HSCT Clinical, Electrocardiographic, and Echocardiographic Parameters in Multiple Myeloma and Non–Multiple Myeloma Patients

	**MM**		**Non-MM**			
	**Preop**	**Postop**	**Preop**	**Postop**	**p-value**	**ES (r)**
**BMI, kg/m^2**	26.75 (17.60 : 29.40)	25.30 (16.20 : 28.10)	25.85 (18.20 : 31.20)	24.25 (19.30 : 29.40)	0.912	0.02
**ECG-Heart Rate, bpm**	76 (62 : 93)	73.50 (56 : 95)	83.50 (59 : 98)	87.50 (66 : 108)	0.762	0.07
**ECG-P wave duration, ms**	115 (99 : 120)	118 (79 : 157)	108(100 : 114)	110 (93 : 118)	**0.041**	0.46
**ECG-PR duration, ms**	155 (116 : 193)	145.50 (111 :180)	154 (128 : 172)	153 (129 : 172)	0.796	0.06
**ECG-QRS duration, ms**	91 (73 : 112)	93 (69 : 108)	91 (63 : 102)	84 (63 : 96)	0.940	0.02
**ECG-QT duration, ms**	378 (358 : 392)	376.50 (353 :394)	385 (329 : 420)	367.50(327 : 393)	0.623	0.11
**ECG-QTc duration, ms**	406 (395 : 433)	400 (384 :420)	403.50 (395 : 432)	409.50 (388 : 422)	0.820	0.05
**ECG-RV_5–SV_1, voltage**	2.14 (0.26 : 2.76)	2.43 (1.06 : 3.04)	1.56(0.43 : 4.74)	1.85 (1.10 : 3.77)	0.353	0.21
**Systolic Blood Pressure, mmHg**	127 (106 : 145)	127.50 (103 : 138)	123 (90 : 137)	121.50 (97 : 128)	0.241	0.26
**Diastolic Blood Pressure, mmHg**	73 (65 : 98)	76 (69 : 96)	80 (60 : 99)	78 (65 : 97)	0.880	0.03

**LVIDd, mm**	44.50 (39 : 53)	43.50 (36 : 51)	47 (40 : 54)	45.50 (34 : 53)	0.172	0.31
**LVIDs, mm**	31 (24 : 35)	29.50 (25 : 35)	32 (26 : 41)	29 (23 : 41)	0.401	0.19
**SV, mL**	52.02 (42.31 : 84.21)	48.82 (32.47 : 69.56)	64.66 (40.41 : 76.72)	59.91 (26.15 :80.54)	0.105	0.36
**EF, %**	61.80 (51.60 : 68.80)	58.70 (51.20 : 63.60)	61.05 (48 : 71.70)	62.60 (45.20 : 71.20)	0.820	0.05
**LV Mass (Ass_c), g**	137.50 (98 : 161)	125 (102 : 167)	145 (82 : 219)	133.50 (67 : 184)	0.970	0.01
**LV Mass Index, g/m^2**	73.30 (56.40 : 83.80)	69.30 (58.60 : 96)	76.05 (48.60 : 115.90)	70.45 (40.20 : 96.40)	0.739	0.07
**LV Mass Index (ht) ,g/m^2**	80 (61.20 : 94.60)	74.15 (63.70 : 89.30)	87.20 (48.20 : 121.70)	79.40 (39.60 : 102)	0.739	0.07
**LV Mass (Penn), g**	157.50 (108 : 187)	141.50 (113 : 195)	166.50 (88 : 260)	153 (69 : 215)	1.000	0
**EF (BP), %**	62.05 (55.40 : 65.60)	60.45 (54.70 : 64.50)	60.20 (51.10 : 67.70)	61.05 (54.20 :65.90)	0.496	0.15
**EF (A4C), %**	62.55 (54.90 : 65.80)	58.90 (51.30 : 67.40)	59.90 (50.80 : 69.70)	61.05 (51.20 : 65.20)	0.256	0.25
**EF (A2C), %**	62.50 (55.10 : 66.10)	59.80 (56.30 : 70.30)	61.30 (50.30 : 67.50)	62.25 (56.20 : 66.50)	0.762	0.07
**GLS endo peak avg, %**	-19.30 (-21.80 : -17.10)	-19.55 (-21.50 : -17.10)	-20.05 (-22.30 : -13.60)	-18.75 (-22.50 : -14.50)	0.791	0.06

**GLS endo peak (A4C), %**	-19.90 (-22.80 : -16.70)	-19.10 (-23.20 : -16.20)	-21.70 (-24 : -15.10)	-19.25 (-23.70 : -14.40)	0.436	0.17
**GLS endo peak (A2C), %**	-19.50 (-21.80 : -16.30)	-19.85 (-21.60 : -17.20)	-17.85 (-23.90 : -13.20)	-19.85 (-21.40 : -14.60)	0.326	0.22
**GLS endo peak (A3C), %**	-19.25 (-24 : -16.60)	-19.15 (-24.30 : -17)	-19.30 (-22.90 : -12.60)	-18.80 (-22.60 : -14.40)	0.850	0.04
**AutoGLS endo (A4C), %**	-20.90 (-23.50 : -16.10)	-19.60 (-22.10 : -14.80)	-22.40 (-25.10 : -14.60)	-19.70 (-23.70 : -14)	0.414	0.19

Table S1. (Continued)

	**MM**		**Non MM**			
	**Preop**	**Postop**	**Preop**	**Postop**	**p-value**	**ES (r)**
**AutoGLS endo (A2C), %**	-19.40 (-22.80 : -16.90)	-20.15 (-21.90 : -16.50)	-21.50 (-24.20 : -14.80)	-21.30 (-23.10 : -15.60)	0.870	0.04
**AutoGLS endo (A3C), %**	-17.85 (-24.90 : -14.30)	-20.60 (-23.60 : -11.90)	-20 (-22.90 : -13.90)	-18.90 (-23.40 : -14.80)	0.235	0.27
**AutoGLS endo (Median), %**	-19.30 (-23.70 : -17.20)	-19.60 (-21.90 : -16.20)	-20.20 (-23 : -14.80)	-19.50 (-22.80 : -14.80)	0.513	0.15

**AutoGLS myo (A4C), %**	-18.20 (-20.50 : -14.10)	-17.20 (-18.90 : -12.50)	-19.40 (-21.80 : -12.70)	-16.70 (-20.70 : -11.90)	0.438	0.18
**AutoGLS myo (A3C), %**	-16.90 (-19.90 : -14.60)	-17 (-18.40 : -8.80)	-18.60 (-21.40 : -12.90)	-16.10 (-19.10 : -11.90)	0.515	0.15
**AutoGLS myo (A2C), %**	-14.70 (-19.10 : -12)	-17.70 (-19.10 : -14.20)	-16.70 (-19.10 : -11.60)	-18.70 (-20.50 : -13)	0.326	0.23
**AutoGLS myo (Median), %**	-16.50 (-19.60 : -14.70)	-17.15 (-18.20 : -13.40)	-17.60 (-20.10 : -12.40)	-16.75 (-19.50 : -12.30)	0.505	0.16
**Auto EDV (BP), mL**	116.89 (96.35 : 159.14)	125.69 (69.09 : 152.87)	124.91 (75.03 : 180.01)	116.31 (77.50 : 190.40)	0.905	0.03
**Auto ESV (BP), mL**	42.52 (34.62 : 53.80)	47.30 (28.88 : 64.95)	46.71 (27.27 : 74.54)	37.86 (24.64 : 70.74)	0.968	0.01
**Auto EDVI (BP), mL/m^2**	65.19 (54.89 : 79.45)	69.13 (39.74 : 81.61)	64.44 (47.14 : 107.70)	60.66 (48.27 : 101.59)	1.000	0
**Auto ESVI (BP), mL/m^2**	21.80 (19.19: 28.63)	27.04 (16.61 : 32.04)	24.10 (17.13 : 47.71)	20.53 (15.35 : 44.03)	0.780	0.06
**Auto SV (BP), mL**	78.91 (59.79 : 106.33)	75.31 (40.21 : 102.21)	78.97 (47.76 : 110.64)	74.89 (49.80 : 120.46)	0.905	0.03
**Auto EF (BP), %**	65.25 (53.90 : 69.20)	60.50 (54.40 : 68.50)	62.80 (55.70 : 69.70)	64.40 (56.70 : 69.40)	0.497	0.16
**Mitral E wave rate, m/s**	0.67 (0.46 : 0.97)	0.63 (0.43 : 0.81)	0.72 (0.34 : 1.12)	0.70 (0.44 : 0.84)	0.762	0.07

**Mitral A wave rate, m/s**	0.86 (0.66 : 1.24)	0.76 (0.55 : 1.12)	0.76 (0.60 : 0.94)	0.71 (0.62 : 0.92)	0.140	0.33
**Mitral Deceleration Slope, m/s^2**	3.67 (2.57 : 44.20)	3.43 (2.05 : 4.26)	4.24 (1.41 : 7.68)	3.67 (1.93 : 30.30)	0.971	0.01
**Mitral E/A ratio**	0.81 (0.48 : 1.36)	0.77 (0.63 : 1.15)	0.94 (0.56 : 1.48)	0.90 (0.71 : 1.25)	0.427	0.18
**Mitral A/E ratio**	1.23 (0.74 : 2.06)	1.29 (0.87 : 1.59)	1.28 (0.68 : 1.79)	1.11 (0.80 : 1.42)	0.653	0.1
**Mitral Deceleration Time, ms**	174.50 (141 : 198)	181 (150 : 229)	188.50 (139 : 249)	189 (141 : 226)	0.597	0.12
**E wave duration,, ms**	194 (97 : 294)	208.50 (140 : 264)	228 (121 : 288)	248 (162 :296)	0.256	0.25
**A wave duration, ms**	132 (94 : 159)	135 (110 : 194)	128 (105 : 137)	129 (119 : 175)	0.323	0.22
**Mitral R–R interval, ms**	714 (579 :943)	852.50 (647 : 1158)	784 (579 : 924)	786.50 (679 : 916)	0.364	0.2
**IVRT (MV), ms**	69.50 (35 : 115)	61.50 (43 : 97)	60 (46 : 89)	76 (59 : 117)	1.000	0
**Mitral CO duration, ms**	365 (315 : 415)	404 (348 : 447)	392 (326 : 450)	396 (350 : 436)	0.121	0.35
**LVET (Ao), ms**	256 (221 : 285)	270.50 (213 : 307)	254.50 (228 :299)	262.50 (226 : 296)	0.733	0.08
**LV MPI**	0.48 (0.14 : 0.73)	0.54 (0.31 : 0.77)	0.45 (0.33 : 0.86)	0.54 (0.29 : 0.68)	0.791	0.06

Table S1. (Continued)

	MM		Non MM			
	**Preop**	**Postop**	**Preop**	**Postop**	**p-value**	**ES (r)**
**TAPSE, mm**	23.50 (20 : 28)	22 (12 : 24)	25 (15 : 27)	23 (18 : 31)	0.561	0.14
**RV FAC, %**	42.60 (30 : 60)	42.40 (29.70 : 50.90)	52.70 (44.40 : 57.30)	48 (35.60 : 57.10)	**0.041**	0.46
**RV freewall strain (FWSL), %**	-25.30 (-35.80 : -23.20)	-24.40 (-29.10 : -23.20)	-29.35 (-34.60 : -22.70)	-28.60 (-32.20 : -23.70)	0.165	0.31
**RV 4C strain, %**	-21.75 (-28.10 : -18.10)	-22.45 (-25.90 : -18.40)	-24.20 (-30.60 : -20.40)	-23.60 (-26.80 : -19)	0.198	0.29
**LALs (A4C), mm**	49.50 (38 : 58)	48 (43 : 53)	48.50 (40 : 58)	46 (36 : 53)	0.849	0.04
**LA volume(A4C), mL**	42.01 (18.70 : 65.82)	36.82 (27.61 : 53.10)	40.51 (17.81 : 85.33)	46.86 (16.66 : 63.94)	0.520	0.14
**LA volume index (A4C), mL/m^2**	22.26 (9.89 : 32.86)	20.70 (13.75 : 30.45)	19.94 (11.02 : 48.19)	25.64 (11.06 : 39.79)	0.529	0.14
**LALs (A2C), mm**	51.50 (26 : 62)	49.50 (39 : 56)	47 (38 : 61)	47 (34 : 59)	0.970	0.01
**LA volume (A2C), mL**	43.74 (19.56 : 73.47)	48.20 (25.28 : 81.68)	44.12 (27.21 : 78.40)	51.26 (16.49 : 91.57)	0.971	0.01

**LA volume index (A2C), mL/m^2**	23.76 (10.76 : 38.28)	23.90 (14.54 : 43.61)	23.67 (16.84 : 44.28)	27.32 (10.95 : 43.12)	0.971	0.01
**LA volume (BP), mL**	46.39 (28.77 : 67)	43.27 (30.71 : 63.19)	40.88 (22.38 : 84.21)	53.52 (17.48 : 75.80)	0.529	0.14
**LA volume index (BP), mL/m^2**	24.51 (14.83 : 33.45)	23.47 (17.66 : 33.74)	24.74 (13.85 : 47.56)	28.77 (11.61 : 38.47)	0.739	0.07
**LAAs (A4C), cm^2**	16.66 (9.82 : 21.92)	15.47 (12.35 : 18.58)	15.84 (9.72 : 24.81)	16.55 (9.19 : 19.82)	0.436	0.17
**LAAs (A2C), cm^2**	16.91 (8.32 : 22.98)	17.18 (12.04 : 23.87)	15.75 (11.94 : 24.55)	16.99 (8.44 : 25.67)	0.853	0.04
**LA dia–sys, mm**	3.45 (2 : 5)	3.45 (2 : 5)	3.20 (2 : 4)	3.10 (2 : 4)	0.187	0.21
**LA sr (ED), 1/s**	55.75 (32.10 : 66.70)	40 (31.40 : 73.10)	46.10 (26.30 : 62)	42.90 (12.30 : 64.20)	0.075	0.40
**LA Scd (ED), 1/s**	-25.65 (-313 : -19.60)	-20.80 (-41 : -10.70)	-21.10 (-37.60 : -17.50)	-21 (-36.70 : -15.40)	0.143	0.33
**LA Sct (ED), 1/s**	-26.45 (-38.50 : -12.50)	-21.60 (-33.20 : -10.30)	-22.90 (-36.20 : -7)	-20.50 (-35.30 : -12.50)	0.218	0.28
**LA sr (AC), 1/s**	42.60 (28.50 : 53)	33.65 (27.40 : 54.80)	37.05 (24.60 : 49.90)	35.20 (25.70 : 47.40)	0.063	0.42
**LA Scd (AC), 1/s**	-20.70 (-34.10 : -14.70)	-17.40 (-33.10 : -8.60)	-17.60 (-31 : -12.90)	-17.45 (-32.50 : -13.50)	0.364	0.20
**LA Sct (AC), 1/s**	-21 (-27.70 : -11.10)	-17.70 (-24.90 : -9.30)	-18.65 (-26.50 : -6.60)	-17.05 (-26.10 : -11.10)	0.212	0.28

**RA diametre (lax), mm**	50.50 (44 : 57)	48 (40 : 58)	49.50 (41 : 60)	47 (32 : 60)	0.820	0.05
**e' (MV anulus septal), cm/s**	8.28 (6.12 : 11.59)	8.50 (7.22 : 10.08)	9.26 (7.26 : 15.64)	9.54 (7.26 : 12.28)	0.218	0.28
**a' (MV anulus septal), cm/s**	12.64 (9.50 : 16.33)	11.67 (8.17 : 16.03)	11.11 (7.31 : 14.86)	10.64 (7.47 : 14.84)	**0.029**	0.49
**e'/a' (MV anulus septal)**	0.67 (0.40 : 1.16)	0.80 (0.45 : 1.20)	0.82 (0.52 : 1.83)	0.81 (0.65 : 1.37)	0.075	0.40
**E/e' (MV anulus septal)**	8.50 (5.74 : 10.66)	7.27 (6.01 : 8.68)	6.76 (4.73 : 11.69)	6.72 (5.46 : 9.56)	0.218	0.28

Table S1. (Continued)

	**MM**		**Non- MM**			
	**Preop**	**Postop**	**Preop**	**Postop**	**p-value**	**ES (r)**
**E/e' median (MV anulus), cm/s**	6.84 (4.28 : 9.13)	6.11 (5.29 : 7.61)	6.30 (3.68 : 7.22)	6.12 (5.02 : 8.70)	0.436	0.17
**S' septal, cm/s**	11.05 (7.61 : 12.80)	9.09 (7.65 : 11.43)	9.20 (7.19 : 11.05)	9.23 (6.14 : 12.55)	0.052	0.43
**e' lateral (MV anulus), cm/s**	12.05 (9.17 : 14.36)	11.88 (9.20 : 13.97)	13.69 (8.54 : 22.55)	10.94 (7.68 : 22.81)	0.143	0.33
**a' lateral (MV anulus), cm/s**	13.88 (8.05 : 19.26)	10.88 (9.37 : 16.75)	9.84 (7.94 : 18.90)	12.02 (6.28 : 21.09)	**0.038**	0.47
**e'/a' lateral (MV anulus)**	0.78 (0.61 : 1.70)	1.06 (0.61 : 1.41)	1.48 (0.54 : 2.66)	0.81 (0.48 : 2.39)	**0.045**	0.45

**E/e' lateral (MV anulus)**	6.17 (3.41 : 8.20)	5.06 (4.56 : 6.77)	5.33 (3.02 : 6.55)	5.67 (3.68 : 8.20)	0.199	0.29
**S' lateral, cm/s**	10.71 (7.98 : 15.12)	11.10 (7.44 : 13.81)	12.09 (7.19 : 15.12)	10.02 (6.86 : 13.68)	0.326	0.22
**RV e' lateral, cm/s**	12.28 (8.19 : 18.02)	9.52 (7.42 : 17.88)	12.47 (6.19 : 18.17)	12.15 (8.48 : 18.70)	0.971	0.01
**RV a' lateral, cm/s**	17.98 (13.79 : 23.38)	15.04 (10.02 : 21.83)	17.59 (8.98 : 24.98)	16.12 (10.61 : 20.35)	0.684	0.09
**RV s' lateral, cm/s**	16.09 (12.05 : 17.71)	13.75 (7.90 : 23.44)	14.11 (13.25 : 16.45)	14.78 (8.18 : 19.73)	0.063	0.41
**SI**	27.80 (24.21 : 41.31)	26.91 (18.67 : 34.42)	35.11 (24.08 : 43.63)	33.16 (15.72 : 45.29)	**0.023**	0.51

ES: Effect Size. Descriptive summaries are reported as median (minimum : maximum). Baseline comparisons between groups (pre-op) were performed using the Mann-Whitney U test. Effect sizes were computed as $r = |Z|/\sqrt{n}$ *(where Z is the standardized test statistic and n is the total sample size; r is reported as absolute magnitude). Interpretation thresholds (Cohen's guidelines): small 0.10, medium 0.30, large 0.50+.*

Abbreviations:

A2C, apical two-chamber; A3C, apical three-chamber; A4C, apical four-chamber; BMI, body mass index; BP, blood pressure; bpm, beats per minute; ECG, electrocardiography; ED, end-diastole; AC, atrial contraction; EF, ejection fraction; EDV/ESV, end-diastolic/end-systolic volume; EDVI/ESVI, end-diastolic/end-systolic volume index; GLS, global longitudinal strain; endo, endocardial; myo, myocardial; IVRT, isovolumetric relaxation time; LA, left atrium; **LALs**, left atrial systolic major-axis length (A4C/A2C); LAAs, left atrial end-systolic area (A4C/A2C); LA Vol, left atrial end-systolic volume; LASr/LAScd/LASct, left atrial

reservoir/conduit/contraction strain (reference ED or AC); LV, left ventricle; LVIDd/LVIDs, LV internal diameter end-diastole/end-systole; LVET, LV ejection time; MPI, myocardial performance index; MV, mitral valve; C–O dur, mitral valve closure-to-opening duration; MM, multiple myeloma; Non-MM, non–multiple myeloma; RA, right atrium; RV, right ventricle; FAC, fractional area change; TAPSE, tricuspid annular plane systolic excursion; SV, stroke volume.

Supplementary Table S2. Full Longitudinal Analysis of Clinical and Echocardiographic Parameters in Multiple Myeloma and Non–Multiple Myeloma Patients: Main Effects of Time, Group, and Time × Group Interaction (ART Model)

	**Group**			**Time**			**Group x Time**		
	**p-value**	**q-value**	**ES (η_p^2)**	**p-value**	**q-value**	**ES (η_p^2)**	**p-value**	**q-value**	**ES (η_p^2)**
**BMI, kg/m²**	0.807	0.807	0	0.008	**0.025**	0.33	0.745	0.794	0.01
**ECG Heart Rate, bpm**	0.251	0.715	0.07	0.430	0.501	0.03	0.441	0.631	0.03
**ECG P duration, ms**	0.006	**0.043**	0.35	0.245	0.344	0.07	0.441	0.631	0.03
**ECG PR duration, ms**	0.927	0.927	0	0.904	0.904	0	0.699	0.815	0.01
**ECG QRS duration, ms**	0.618	0.866	0.01	0.089	0.310	0.15	0.413	0.631	0.04
**ECG QT duration, ms**	0.82	0.927	0	0.134	0.312	0.12	0.129	0.631	0.12
**ECG QTc duration, ms**	0.437	0.765	0.03	0.243	0.344	0.07	0.451	0.631	0.03
**ECG RV_5–SV_1, voltage**	0.306	0.715	0.06	0.015	0.104	0.29	0.867	0.867	0
**Systolic Blood Pressure, mmHg**	0.216	0.649	0.08	0.189	0.284	0.09	0.660	0.794	0.01
**Diastolic Blood Pressure, mmHg**	0.724	0.807	0.01	0.941	0.941	0	0.794	0.794	0

**LVIDd, mm**	0.485	1.000	0.03	0.022	0.240	0.26	0.115	0.350	0.13
**LVIDs, mm**	0.847	1.000	0	0.217	0.412	0.08	0.047	0.254	0.20
**SV, mL**	0.272	1.000	0.07	0.025	0.240	0.25	0.508	0.742	0.02
**EF, %**	0.475	1.000	0.03	0.718	0.870	0.01	0.359	0.620	0.05
**LV Mass (Ass_c), g**	0.908	1.000	0	0.127	0.320	0.12	0.700	0.839	0.01
**LV Mass Index, g/m^2**	0.800	1.000	0	0.135	0.320	0.12	0.795	0.839	0
**LV Mass Index (ht), g/m^2**	0.664	1.000	0.01	0.110	0.320	0.14	0.784	0.839	0
**LV Mass (Penn), g**	0.885	1.000	0	0.130	0.320	0.12	0.663	0.839	0.01
**EF (BP), %**	0.983	1.000	0	0.588	0.869	0.02	0.171	0.367	0.1
**EF (A4C), %**	0.914	1.000	0	0.130	0.320	0.12	0.067	0.254	0.17
**EF (A2C), %**	0.722	1.000	0.01	0.732	0.870	0.01	0.398	0.630	0.04
**GLS endo peak avg, %**	1.000	1.000	0	0.419	0.719	0.04	0.659	0.790	0.01
**GLS endo peak (A4C), %**	0.566	0.869	0.02	0.065	0.195	0.18	0.590	0.790	0.02

Table S2. (Continued)

	**Group**			**Time**			**Group x Time**		
	**p-value**	**q-value**	**ES (η_p^2)**	**p-value**	**q-value**	**ES (η_p^2)**	**p-value**	**q-value**	**ES (η_p^2)**
**GLS endo peak (A2C), %**	0.429	0.869	0.04	0.285	0.571	0.06	0.453	0.790	0.03
**GLS endo peak (A3C), %**	0.526	0.869	0.02	0.734	0.734	0.01	0.604	0.790	0.02
**AutoGLS endo (A4C), %**	0.662	0.869	0.01	0.021	0.086	0.27	0.517	0.790	0.03
**AutoGLS endo (A2C), %**	0.508	0.869	0.03	0.718	0.734	0.01	0.790	0.790	0
**AutoGLS endo (A3C), %**	0.718	0.869	0.01	0.509	0.727	0.03	0.087	0.790	0.16

**AutoGLS endo (Median), %**	0.613	0.869	0.02	0.563	0.727	0.02	0.319	0.790	0.06
**AutoGLS myo (A4C), %**	0.725	0.869	0.01	0.008	**0.045**	0.35	0.598	0.790	0.02
**AutoGLS myo (A3C), %**	0.796	0.869	0	0.086	0.207	0.17	0.185	0.790	0.11
**AutoGLS myo (A2C), %**	0.279	0.869	0.07	0.001	**0.013**	0.47	0.772	0.790	0.01
**AutoGLS myo (Median),%**	0.554	0.869	0.02	0.606	0.727	0.02	0.393	0.790	0.05
**Auto EDV (BP), mL**	0.75	1.000	0.01	0.911	0.944	0	0.174	0.367	0.11
**Auto ESV (BP), mL**	0.514	1.000	0.03	0.944	0.944	0	0.020	0.187	0.28
**Auto EDVI (BP), mL/m^2**	0.509	1.000	0.03	0.644	0.870	0.01	0.129	0.350	0.13
**Auto ESVI (BP), mL/m^2**	0.416	1.000	0.04	0.483	0.835	0.03	0.009	0.162	0.34
**Auto SV (BP), mL**	1.000	1.000	0	0.828	0.926	0	0.733	0.839	0.01
**Auto EF (BP), %**	0.59	1.000	0.02	0.595	0.869	0.02	0.058	0.254	0.2
**Mitral E rate, m/s**	0.534	0.808	0.02	0.403	0.654	0.04	0.886	0.967	0
**Mitral A rate, m/s**	0.152	0.663	0.11	0.076	0.453	0.16	0.303	0.828	0.06
**Mitral Deceleration, Slope, m/s^2**	0.788	0.946	0	0.763	0.833	0.01	0.483	0.828	0.03
**Mitral E/A ratio**	0.166	0.663	0.1	0.988	0.988	0	0.988	0.988	0
**Mitral A/E ratio**	0.287	0.808	0.07	0.663	0.833	0.01	0.843	0.967	0
**Mitral Deceleration Slope ,m/s^2**	0.518	0.808	0.02	0.711	0.833	0.01	0.422	0.828	0.04
**E wave duration, ms**	0.034	0.408	0.23	0.234	0.561	0.08	0.732	0.967	0.01

Table S2. (Continued)

	**Group**			**Time**			**Group x Time**		
	**p-value**	**q-value**	**ES (η_p^2)**	**p-value**	**q-value**	**ES (η_p^2)**	**p-value**	**q-value**	**ES (η_p^2)**
**A wave duration,ms**	0.611	0.814	0.01	0.281	0.562	0.06	0.707	0.967	0.01

**Mitral R–R interval, ms**	0.965	0.965	0	0.116	0.463	0.13	0.181	0.828	0.1
**IVRT (MV), ms**	0.497	0.808	0.03	0.436	0.654	0.03	0.352	0.828	0.05
**Mitral CO duration, ms**	0.538	0.808	0.02	0.019	0.232	0.27	0.064	0.770	0.18
**LVET (Ao), ms**	0.909	0.965	0	0.164	0.493	0.1	0.282	0.828	0.06
**LV MPI**	0.712	1.000	0.01	0.210	0.412	0.09	0.947	0.947	0
**TAPSE, mm**	0.592	0.835	0.02	0.310	0.413	0.07	0.223	0.767	0.1
**RV FAC, %**	0.006	**0.049**	0.35	0.111	0.222	0.14	0.641	0.855	0.01
**RV free-wall strain (FWSL), %**	0.028	0.114	0.24	0.233	0.372	0.08	0.946	0.968	0
**RV 4C strain, %**	0.224	0.598	0.08	0.860	0.860	0	0.384	0.767	0.04
**LALs (A4C), mm**	0.432	0.923	0.03	0.254	0.553	0.07	0.710	0.755	0.01
**LA volume(A4C), mL**	0.834	0.923	0	0.683	0.836	0.01	0.355	0.543	0.05
**LA volume index(A4C), mL/m^2**	0.798	0.923	0	0.663	0.836	0.01	0.383	0.543	0.04
**LALs (A2C), mm**	0.593	0.923	0.02	0.927	0.985	0	0.517	0.618	0.02
**LA volume (A2C), mL**	0.874	0.923	0	0.695	0.836	0.01	0.369	0.543	0.05
**LA volume index (A2C), mL/m^2**	0.709	0.923	0.01	0.738	0.836	0.01	0.376	0.543	0.04
**LA volume (BP), mL**	0.920	0.923	0	0.620	0.836	0.01	0.204	0.543	0.09
**LA volume index (BP), mL/m^2**	0.853	0.923	0	0.386	0.730	0.04	0.240	0.543	0.08
**LAAs (A4C), cm^2**	0.602	0.923	0.02	1.000	1.000	0	0.500	0.618	0.03
**LAAs (A2C), cm^2**	0.923	0.923	0	0.681	0.836	0.01	0.545	0.618	0.02
**LA dia–sys, mm**	0.703	0.923	0.01	0.260	0.553	0.07	1.000	1.000	0

LA sr (ED), 1/s	0.359	0.923	0.05	0.002	**0.018**	0.42	0.042	0.356	0.21

Table S2. (Continued)

	**Group**			**Time**			**Group x Time**		
	**p-value**	**q-value**	**ES (η_p^2)**	**p-value**	**q-value**	**ES (η_p^2)**	**p-value**	**q-value**	**ES (η_p^2)**
**LA Scd (ED) , 1/s**	0.396	0.923	0.04	0.174	0.553	0.1	0.180	0.543	0.1
**LA Sct (ED) , 1/s**	0.435	0.923	0.03	0.102	0.434	0.14	0.211	0.543	0.09
**LA sr (AC) , 1/s**	0.521	0.923	0.02	0.002	**0.018**	0.43	0.007	0.115	0.34
**LA Scd (AC) , 1/s**	0.639	0.923	0.01	0.205	0.553	0.09	0.308	0.543	0.06
**LA Sct (AC) , 1/s**	0.434	0.923	0.03	0.098	0.434	0.14	0.239	0.543	0.08
**RA diametre (lax), mm**	0.791	0.870	0	0.080	0.213	0.16	0.968	0.968	0
**e' (MV anulus septal), cm/s**	0.125	0.509	0.13	0.951	0.951	0	0.876	0.876	0
**a' (MV anulus septal), cm/s**	0.183	0.509	0.1	0.102	0.266	0.14	0.179	0.245	0.1
**e'/a' (MV anulus septal)**	0.104	0.509	0.14	0.295	0.541	0.06	0.106	0.167	0.14
**E/e' (MV anulus septal), cm/s**	0.248	0.546	0.07	0.456	0.717	0.03	0.396	0.436	0.04
**E/e' median (MV anulus)**	0.498	0.685	0.03	0.673	0.925	0.01	0.243	0.297	0.07
**S' septal, cm/s**	0.185	0.509	0.1	0.066	0.241	0.18	0.068	0.143	0.17

**e' lateral (MV anulus), cm/s**	0.589	0.719	0.02	0.043	0.237	0.21	0.025	0.136	0.25
**a' lateral (MV anulus), cm/s**	0.397	0.624	0.04	0.921	0.951	0	0.052	0.142	0.19
**e'/a' lateral (MV anulus)**	0.299	0.547	0.06	0.026	0.237	0.25	0.003	**0.031**	0.40
**E/e' lateral (MV anulus)**	0.846	0.930	0	0.762	0.931	0.01	0.049	0.142	0.20
**S' lateral, cm/s**	0.966	0.966	0	0.121	0.266	0.13	0.078	0.143	0.16
**RV e' lateral, cm/s**	0.626	0.835	0.01	0.573	0.654	0.02	0.343	0.767	0.05
**RV a' lateral, cm/s**	0.870	0.870	0	0.034	0.184	0.23	0.617	0.855	0.01
**RV s' lateral, cm/s**	0.608	0.835	0.01	0.046	0.184	0.20	0.061	0.489	0.18
**SI**	0.157	1.000	0.11	0.114	0.320	0.13	0.277	0.526	0.07

ES: Effect Size. Main effects of Group, Time, and their Interaction were analyzed using the non-parametric Aligned Rank Transform (ART) test. To control for multiple comparisons, p-values for main analyses were adjusted using the Benjamini-Hochberg (FDR) method; p-value represents the nominal (raw) p-value, and q-value .represents the adjusted p-value. Effect sizes were computed as Partial Eta Squared ($\boldsymbol{\eta_p^2}$). Interpretation thresholds (Cohen's guidelines): small 0.01, medium 0.06, large ≥0.14. Bold values indicate statistical significance ($p < 0.05$).

Abbreviations:

AC, atrial contraction; ART, aligned rank transform; A2C/A3C/A4C, apical two-/three-/four-chamber; BMI, body mass index; BP, blood pressure; bpm, beats per minute; ECG, electrocardiography; ED, end-diastole; EF, ejection fraction; EDV/ESV, end-diastolic/end-systolic volume; EDVI/ESVI, end-diastolic/end-systolic volume index; FDR, false discovery rate; GLS, global longitudinal strain; endo, endocardial; myo, myocardial; IVRT, isovolumetric

relaxation time; LA, left atrium; **LALs**, left atrial systolic major-axis length (A4C/A2C); **LAAs**, left atrial end-systolic area (A4C/A2C); LA Vol, left atrial end-systolic volume; **LASr/LAScd/LASct**, left atrial reservoir/conduit/contraction strain (reference ED or AC); LV, left ventricle; LVIDd/LVIDs, LV internal diameter at end-diastole/end-systole; LVET, LV ejection time; MPI, myocardial performance index; MV, mitral valve; **C–O duration**, mitral valve closure-to-opening duration; MM, multiple myeloma; Non-MM, non–multiple myeloma; RA, right atrium; RV, right ventricle; FAC, fractional area change; TAPSE, tricuspid annular plane systolic excursion; SI, stroke index
